# The effectiveness of the trans-theoretical model in managing adult obese and overweight individuals

**DOI:** 10.12669/pjms.40.4.7373

**Published:** 2024

**Authors:** Cigdem Gereklioglu, Kenan Topal, Hatice Velipasaoglu, Huseyin Aksoy

**Affiliations:** 1 Cigdem Gereklioglu Associate Professor, Cukurova University Medical School, Department of Family Medicine, Adana/Turkey; 2 Kenan Topal Associate Professor, Health Sciences University, Adana City, Training and Research Hospital, Department of Family Medicine, Adana/Turkey; 3 Hatice Velipaşaoglu Huseyin Aksoy, Health Sciences University, Adana City, Training and Research Hospital, Department of Family Medicine, Adana/Turkey; 4 Huseyin Aksoy, Health Sciences University, Adana City, Training and Research Hospital, Department of Family Medicine, Adana/Turkey

**Keywords:** Transtheoretical model, Obesity, Overweight, Diabetes mellitus, Type-2, Weight loss, Glycemic control

## Abstract

**Background & Objective:**

The prevalence of obesity is gradually increasing in our country and worldwide. Being obese and overweight are risk factors for chronic diseases. Obesity has a multifactorial etiology, so treatment should involve lifestyle changes, psychological strategies, pharmacologic treatment, and bariatric surgery. The present study aimed to investigate the effectiveness of the trans-theoretical stages of change (TTM SOC) model in managing adult obese and overweight patients.

**Methods:**

This prospective cohort study was conducted with 133 adults who were admitted to the Family Medicine Outpatient Clinic of Adana City Research and Training Hospital between April 1, 2017, and April 30, 2019. Socio-demographic characteristics, blood pressures, anthropometric measurements, and laboratory data were compared between the baseline and the first, third, and sixth months.

**Results:**

Body mass index (BMI) was higher among those with a low educational level. The mean age, the number of medications used, and the metabolic parameter values were significantly lower among the participants who did not have a chronic disease. Blood pressures, weight, BMI, plasma glucose and insulin, HOMA-IR, and triglyceride were statistically significantly higher at the baseline compared to follow-up values. Fasting plasma glucose was higher at the baseline in diabetic patients. The results were compared with Student t and One Way ANOVA tests. The Pearson correlation coefficient was used to demonstrate the association between baseline and repeated metabolic measurements.

**Conclusion:**

The trans-theoretical model is effective in managing adult obese and overweight individuals and also in glycemic control in obese Type-2 diabetics

## INTRODUCTION

Overweight and obesity are defined as “abnormal or excessive fat accumulation that presents a health risk”. While a body mass index (BMI) over 25 is considered overweight, over 30 is considered obese. The prevalence of obesity is gradually increasing in our country and worldwide. According to the World Health Organization (WHO) data, worldwide obesity has nearly tripled since 1975 and in 2016, more than 1.9 billion adults, were overweight and over 650 million were obese. The Global Non-Communicable Disease (NCD) Risk Factor Collaboration reported that if post-2000 trends continue, by 2025, global obesity prevalence will reach 18% in males and surpass 21% in females.^1^-^4^

In Turkiye, the ratio of obesity was reported to be 30.3% (20, 5% in males and 41.0% in females) by the Ministry of Health.^1^-^4^ Kilinc et al. demonstrated that the prevalence of overweight/ obesity in 6-17 years’ school children were 13.2%/4.2% in the southeastern part of Turkiye.^5^ Besides being a global health burden, obesity is also associated with some cancers. The risk of these non-communicable diseases increases even when a person is only slightly overweight and the risk increases as the body mass index increases. Although many of the causative factors are preventable and reversible, no country has yet to reverse the growth of this epidemic.

The main cause of obesity is an imbalance of calories consumed and calories expended besides decreased physical activity due to the changing lifestyles.^1^ Due to the multifactorial etiology of obesity, treatment should involve changes in lifestyle, psychological strategies, pharmacologic treatment, and bariatric surgery. Nevertheless, higher-impact interventions carried out in the primary healthcare setting are needed.^6^ Expanding role of primary health care in obesity management recommends to advance patient motivation, behavior and life style as standard practices.^7^ Ince states that the trans-theoretical model presents the appropriate motivational and teaching strategies for people in different stages of physical activity behaviors.^8^

Despite the strong evidence on the effect of lifestyle changes in the management of obesity and overweight, it may not always be easy for individuals and physicians to achieve goals.^9^ The Transtheoretical Model (TTM) which has long been considered a useful interventional approach in lifestyle modification programs, assumes that behavioral change is complex, unfolding in a sequence of stages. The Stages of Change (SOC) describe the individual’s current intention and engagement toward a targeted health-related behavior. In the first stage (*pre-contemplation*), the individual has no intention to change behavior. In the second stage (*contemplation*), the individual is aware of a problem but has not yet committed to taking action. At the *preparation* stage, the individual has the intention to take action in the next month. At the *action* stage, the individual begins to modify the behavior, and at the last stage (*maintenance*), the new behavior has continued for at least six months.^6^,^9^ The present study aimed to investigate TTM SOC’s effectiveness in managing adult obese and overweight patients.

## METHODS

The study was designed as a prospective cohort study and conducted with 133 obese or overweight adults who were admitted to the Family Medicine Outpatient Clinic of Adana City Research and Training Hospital between April 1, 2017, and April 30, 2019. The sample size was calculated at 126 by using the Epitools program.^10^ Inclusion criteria were being 18 years and above, overweight or obese, able to communicate, being a volunteer for participation, and not having medical or psychiatric problems hindering physical activity.

### Ethical Approval

Ethics committee approval was obtained from the Local Ethics Committee of Health Sciences University (Date: March 28, 2017, Number: 43). Participants’ informed consent was obtained. The study was conducted in accordance with the principles of the Helsinki Declaration and its later amendments.

Data regarding socio-demographic characteristics were collected via a form created by the researchers. Participants’ systolic blood pressure (SBP), diastolic blood pressure (DBP), anthropometric measurements, and laboratory data were recorded. Body mass index was calculated by dividing weight in kilograms by height in meters squared [weight (kg) ÷ height (m^2^)]. Central obesity was defined as a waist circumference of >88 cm in females and >102 cm in males.^11^ Insulin resistance was estimated by using the Homeostatic Model Assessment of Insulin Resistance (HOMA-IR) formula: fasting plasma glucose [mg/dL] X fasting insulin [μu/mL] ÷ 405. HOMA-IR values of ≥2.7 were considered insulin resistance.^12^

### Study Design

Patients’ baseline, first, third, and sixth-month blood pressure, waist circumference, BMI, and metabolic parameters were measured. Patients who were at the second stage of the TTM (*contemplation*) were trained in healthy nutrition and physical activity to be prepared (*preparation*) for the *action* stage. An activity plan of 45 minutes walking daily for five days of the week was done. On the follow-ups in the third and the sixth month, the stages of the behavioral change were checked and their adherence to the diet and physical activity plan was evaluated, and required arrangements were done for the *maintenance* of the behavior. At the first, third, and sixth-month follow-up, total weight lost and metabolic outcomes were recorded. While whole patients came to the first two visits, only 51 (41.3%) came for the sixth-month visit.

### Statistical Analysis

Data were evaluated by using the Statistical Package for Windows version 22,0 (SPSS 22,0). Descriptive statistics were given as mean and standard deviation, frequency, and rates were estimated. Two independent samples t and One Way ANOVA tests were used in inter-group comparisons. The Pearson correlation test was used to determine the correlations between blood pressure, weight, BMI, insulin resistance, and metabolic parameters. A p-level of<0.05 was considered statistically significant.

## RESULTS

The mean age of the participants was 47,3±12,8 years. While 61.7% of them were females, 38.3% were males. The vast majority of the participants were married (82.7%), and 58 (43.6%) had an elementary school education level or below. According to BMI, 23.3% (n=31) of the participants were overweight and 76.7% (n=102) were obese. The mean BMI was higher in females (35, 5±7.6 kg/m^2^) than in males (32.0±4.8 kg/m^2^) (t= -2,942, p=0,004). One-way ANOVA variance analysis showed that height increased and BMI decreased as education level increased (p=0.000 for both). At the baseline, the number of participants who were exercising regularly was 22 (16.5%) and the number of participants who were admitted to a dietitian was 30 (22.6%).

The mean waist circumference was 110. 9±10.0 cm for males (minimum 88- maximum 136 cm) and 110,8 ± 11.9 cm for females (minimum 89- maximum 151 cm). While 41 males (80, 4%) had central obesity, all females had central obesity. The mean blood pressure, anthropometric measurements, and metabolic parameters at the baseline, in the first, third, and sixth months are presented in Table-I. Seventy-nine (59.4%) participants were using at least one medication. The mean number of drugs used was 1.7±2. Comparison of the participants with or without chronic diseases with regard to age, the number of medications used, and metabolic parameters is presented in Table-II.

**Table-I:** The mean blood pressure, anthropometric measurements, and metabolic parameters at the baseline, in the 1^st^, 3^rd^, and 6^th^ months.

	Baseline (n=133), Mean±SD	1^st^ month (n=133) Mean±SD	3^rd^ month (n=133) Mean±SD	6^th^ month (n=51) Mean±SD
Systolic BP (mmHg)	132.3±16.2	127.0±15.7	125.7±12.6	129.5±15.5
Diastolic BP (mmHg)	78.8±10.	77.0±10.2	76.6±9.2	76.5±8.7
Weight (kg)	91.9±16.2	88.0±15.7	84.2±14.9	82.2±15.4
BMI (kg/m^2^)	34,2±6,8	32,9±6,2	31,5±5,9	30,9±5,4
FPG (mg/dL)	122.9±60.9	119.4±49.3	115.3±40.0	112.5±33.7
Fasting insulin (mg/dL)	12.6±7.9	8.9±4.2	8.3±4.1	7.1±3.3
HOMA-IR	3.8±4.0	2.5±1.8	2.3±1.3	1.9±1.0
Triglyceride (mg/dL)	174.6±135.3	138.4±136.5	146.7±121.3	144.0±163.0
HDL (mg/dL)	46.6±11.3	47.3±11.3	48.0±11.8	58.1±70,6

SD: Standard deviation, BP: Blood pressure, BMI: Body mass index, FPG: Fasting plasma glucose, HOMA-IR: Homeostatic model assessment-insulin resistance, HDL: High-density lipoprotein.

**Table-II:** Comparison of the participants with or without chronic diseases with regard to age, number of medications used, and metabolic parameters.

(n=133)	Chronic disease		t*	p

	No (n=50), Mean±SD	Yes (n=83), Mean±SD		
Age (year)	39.4±.5	5.1±10.6	-6.209	0.000^†††^
Number of medications used	0.08±0.3	2.76±2.2	-8.271	0.000^†††^
FPG(mg/dL)	99.0±14.9	137.2±72.7	-3.668	0.000^†††^
Fasting insulin (mg/dL)	12.9±6.3	12.4±8.8	0.362	0.718
HOMA-IR	3.1±1.8	4.3±4.9	-1.998	0.048^†^
Triglyceride (mg/dL)	176.0±175.6	173.8±105.1	0.088	0.930
HDL (mg/dL)	44.1±9.5	48.1±12.1	-2.027	0.045^†^
HbA1c (%)	5.8±0.8	6.9±2.1	-3.621	0.000^†††^
Urea (mg/dL)	23.4±7.8	27.7±9.3	-2.717	0.007^††^

FPG: Fasting plasma glucose, HOMA-IR: Homeostatic model assessment-insulin resistance, HDL: High-density lipoprotein, HbA1c: Hemoglobin A1C, SD: Standard deviation.

* Two independent samples t-test was used,

† p<0.05,

†† p<0,01.

††† p<0,001.

Systolic and diastolic blood pressures, weight, BMI, FPG, plasma insulin, HOMA-IR, and triglyceride were found to be statistically significantly higher at the baseline as compared to the first, third, and sixth-month (p<0.001). HDL values were found to be statistically significantly lower at the baseline as compared to the first and third-month values (p<0.001), however, no significant correlation was detected when evaluated for the sixth-month value (p>0.05).

In diabetic patients (n=39), FPG values were statistically significantly higher at the baseline (179,1±88,5) as compared to the first, third, and sixth-month values (166,4±70,4, 153,0±56,1, 141,8±43,2, respectively). The mean HbA1c values were found to be statistically significantly higher at the baseline (8,3± 2,4) as compared to the first (7,5± 1,8) and third-month (7,1± 1,5) values (r=0,829 – p=0,000, r=0,569 – p=0,000, respectively), however, there was no statistically significant correlation as compared to sixth-month values (7,0±1,2) (r=0,280– p=0,277).

## DISCUSSION

The WHO recommends assisting countries in developing comprehensive public health policies and strategies for improving the prevention and management of obesity. This report draws attention to the importance of diet, exercise, and behavioral approaches.^13^ The present study has revealed that healthy nutrition education and increasing physical activity level through TTM has enabled individuals to lose weight and reduce inflammatory parameters. Similarly, Oğuz et al. reported that a combination of lifestyle changes like diet, and exercise with cognitive behavioral approaches increases the effectiveness of treatment and facilitated maintaining weight loss.^14^ The obesity epidemic environment in Bahrain, especially in school children and youth creates the need for establishing an intervention programme to prevent and control of obesity among these age groups.^15^ Menekli et al. from Türkiye also found that individual consultation prepared in accordance with the transtheoretical model is effective in managing obesity.^16^

Kanter and Cabellero reported that overweight and obesity prevalence in males and females largely varied among countries however, in general, females are more obese than males.^17^ Many studies have found a relationship between socioeconomic status and mean body weight. Molarius et al. associated a low educational status with a higher BMI in almost half of the males and almost all females.^18^ In our study, although the mean weight of males was higher, the mean BMI was higher in females. Also, BMI was significantly higher among those with a low educational level. These results suggested that socioeconomic inequality and low educational status could increase obesity.

Being overweight and obese is strongly associated with a higher incidence of chronic diseases. Keramat et al. reported a higher prevalence of chronic conditions among obese middle-aged and older adults.^19^ Kearns et al. detected diabetes in 42% of females and hypertension in 30% of males in their study which showed that the prevalence of chronic diseases increases with increasing BMI.^20^ In our study, 62.4% of overweight or obese individuals had at least one chronic disease. These results indicate significant clues for improving public health and efforts to reduce obesity would significantly contribute to the prevention of chronic diseases.

The study of de Freitas et al. that investigates the effectiveness of TTM in weight control among obese adult females revealed that at the end of six months, 51 women in the intervention group had a higher weight loss and lower BMI and plasma glucose levels as compared to 35 women in the control group.^6^ A study from Turkiye which conducted the TTM-based motivational interview showed a statistically significantly higher reduction in all metabolic parameters at the end of six months of the intervention period as compared to the control group.^21^ In our study, a statistically significant reduction was detected in SBP, DBP, weight, BMI, FPG, fasting insulin, HOMA-IR, and triglyceride levels, and a statistically significant increase in HDL levels at the first and third months.

Scientific evidence shows that the prevalence of Type 2 DM is three-seven times higher in those who are affected by obesity than in normal-weight adults.^22^ Systematic reviews reported that weight loss corrected glycemic control in diabetic patients.^23^,^24^ So, it may be stated that the most promising approach in the treatment of Type 2 DM is lifestyle changes focused on healthy nutrition and physical activity.^25^ Our study showed that fasting plasma glucose and HBA1c levels significantly decreased at the first, third,^26^ and sixth months and the reduction in HbA1c levels substantially continued at the sixth month.

Primary care institutions have an important role in the prevention of obesity. Even a partial reduction in BMI may have a significant effect on the reduction of chronic diseases. We have obtained promising results in this study which aimed to increase healthy nutrition and physical activity through TTM. However, the final goal should be to enable weight loss and lifestyle changes to be permanent and thereby relieve and reduce the prevalence of chronic diseases. The present study contributes to the literature by drawing attention to behavioral interventions in managing overweight and obesity, an important and gradually increasing public health problem.

### Limitation

The prospective design of the study and six months of follow-up period, most participants’ following the instructions and coming for the first two visits are the strengths of the study however some participants’ not coming for the sixth-month visit may be considered a limitation.

## CONCLUSION

The results of the study showed that TTM-SOC-based intervention is effective in weight loss and correction of metabolic parameters in obese adults and in plasma glucose regulation in obese diabetics. The trans-theoretical model is effective in managing adult obese and overweight individuals.

### Authors Contribution:

**KT** and **CG:** Designed the study.

**HV** and **HA:** Did data collection.

**KT**: Did statistical analysis.

**CG** and **KT:** Did manuscript writing, review and approval of manuscript.

All authors are responsible and accountable for the accuracy or integrity of the work.
